# A Reliable Prognostic Model for HCC Using Histological Grades and the Expression Levels of Related Genes

**DOI:** 10.1155/2021/9512774

**Published:** 2021-10-07

**Authors:** Hao Zhang, Renzheng Liu, Lin Sun, Xiao Hu

**Affiliations:** ^1^Department of Hepatobiliary Pancreatic Surgery, The Affiliated Hospital of Qingdao University, Qingdao, Shandong, China; ^2^Department of ICU, The Affiliated Hospital of Qingdao University, Qingdao, Shandong, China

## Abstract

Hepatocellular carcinoma (HCC) is the most common primary liver malignancy and is a leading cause of cancer-related death worldwide. This study aimed to establish a reliable prognostic model for HCC using histological grades and the expression levels of related genes. The histological grade of a tumor provides prognostic information. The expression data of HCC samples were downloaded from The Cancer Genome Atlas (TCGA) database. We employed the univariate and multivariate Cox regression analyses, as well as the least absolute shrinkage and selection operator (LASSO) regression to establish the prognostic model. After verification of the proposed model using data downloaded from the International Cancer Genome Consortium (ICGC) database, we found that the model was highly reliable, and it was revealed that the prognosis in the high-risk group was significantly worse than that in the low-risk group. Next, we explored the correlation of RiskScore with patients' clinicopathological characteristics, and we found that the RiskScore could be used as an independent prognostic factor, which further confirmed the reliability of our model. In summary, the proposed model could accurately predict the prognosis of HCC patients, assisting clinicians to study the roles of different histological grades of HCC.

## 1. Introduction

Hepatocellular carcinoma (HCC) is the sixth most common type of cancer and the fourth leading cause of cancer-related deaths worldwide [1]. The major risk factors for HCC include viral hepatitis B virus (HBV), hepatitis C virus (HCV), and hepatitis D virus (HDV) and environmental (dietary aflatoxin and iron overload) factors [2–4]. A large number of therapeutic methods have been presented for HCC. Nevertheless, patients' long-term survival remains poor. Thus, there is an urgent need to analyze HCC from a different perspective. Advancements in protein profiling are significant to identify the molecular mechanisms of HCC [5].

Different histological grades affect the treatment and prognosis of HCC patients, highlighting the important role of histological grades [6]. Kurebayashi et al. found that the immune-high subtype was associated with high-grade HCC [7]. Lin et al. demonstrated amide proton transfer-weighted imaging is a significant imaging biomarker, complementing diffusion-weighted imaging for the more accurate and comprehensive characterization of HCC [8]. Ameli et al. reported that volumetric apparent diffusion coefficient and volumetric venous enhancement could predict the grade of tumor differentiation in HCC [9]. Wang et al. found that the expression level of STAT4 was correlated with the histological grade of HCC [10]. Tsai et al. pointed out that a higher expression level of EMMPRIN was significantly associated with the histological grade of HCC [11]. However, few studies have analyzed the differences in gene expression levels in different histological grades of HCC.

In the present study, we collected data from public databases to establish a prognostic model using histological grades of HCC and the expression levels of related genes, and the model could well predict the prognosis of HCC patients, providing new insights into the study of different histological grades of HCC.

## 2. Methods

### 2.1. Data Collection

The expression data (type: FPKM) and clinical data of HCC samples were obtained from The Cancer Genome Atlas (TCGA) database. The test dataset of gene expression and clinical trait data (the Liver Cancer-RIKEN JP) were downloaded from the International Cancer Genome Consortium (ICGC) database. Gene transfer format (GTF) files were downloaded from Ensemble for gene annotation.

### 2.2. Establishment and Validation of the Model

Differentially expressed genes (DEGs) were identified by the “Limma” package in R programming language (ver. 4.0.0). The least absolute shrinkage and selection operator (LASSO) regression with 10-time cross-validation was used to choose the penalty regularization parameter. The coefficient of each gene was forced to shrink to zero, which eliminated the correlation between the selected genes and prevented the model from being overfitting. The data were analyzed by “survival,” “glmnet,” and “survminer” packages in R programming language. The “survivalROC” and “survival” packages were employed to draw receiver operating characteristic (ROC) curves and survival curves, respectively.

### 2.3. Gene Set Enrichment Analysis (GSEA)

GSEA was utilized in this study to compare the differences in survival among different risk-dependent groups in TCGA cohort. An annotated gene set file (c2.cp.kegg.v7.0.symbols.gmt) was selected as the reference. The threshold of *q* value <0.05 was considered as well.

### 2.4. The Analysis of ImmuneScore

The results of deconvolution of tumor-infiltrating immune components were obtained using data collected from the TCGA database that were analyzed by CIBERSORT. The “StromalScore,” “ImmuneScore,” and “ESTIMATEScore” were calculated for each sample by the “estimate” package. The correlations among these indices were analyzed by Spearman's correlation analysis.

## 3. Results

### 3.1. The Prognostic Model in TCGA Cohort

**Figure 1:**
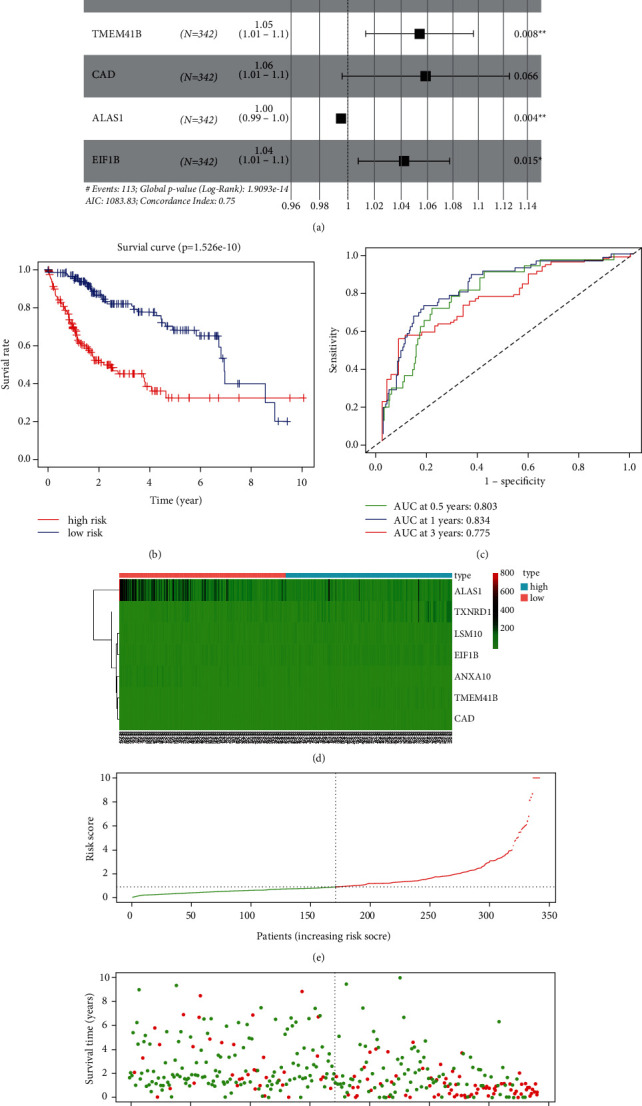
Constructing the prognostic model. (a) The results of multivariate cox regression analysis. (b) Comparison of survival data between the high-risk group and low-risk group. (c) The ROC curves in TCGA cohort. (d) The expression levels of TXNRD1, ANXA10, LSM10, TMEM41B, CAD, ALAS1, and EIF1B in the two groups. (e, f) The survival rates of patients with different RiskScores.

According to patients' age, 371 HCC patients were divided into A (G1-G2) and B (G3-G4) groups. We identified 2308 DEGs in the B group (log fold-change (FC) > 1, *P* value_adj_<0.05). We screened 1340 genes by univariate Cox regression analysis in TCGA cohort. We used LASSO regression and multivariate Cox regression analyses to narrow the number of genes, and finally, 7 genes could be achieved to optimize the model (Figure 1(a)), and the RiskScore of each sample was calculated as follows: RiskScore = TXNRD1^*∗*^0.0104 + ANXA10^*∗*^− 0.0167+LSM10^*∗*^0.0336+TMEM41B^*∗*^0.0526+CAD^*∗*^0.0570+ALAS1^*∗*^ − 0.0046 + EIF1B^*∗*^0.0412. The median RiskScore was used to distinguish high- and low-risk groups. The prognosis in the high-risk group was significantly worse than that in the low-risk group (Figure 1(b)). The values of area under ROC curve (AUC) at 0.5-, 1-, and 3-year survival were 0.803, 0.834, and 0.775, respectively (Figure 1(c)). The heatmap showed that the expression levels of TXNRD1, LSM10, TMEM41B, CAD, and EIF1B in the high-risk group were higher than those in the low-risk group, while opposite results achieved for the expression levels of ANXA10 and ALAS1 (Figure 1(d)). Besides, the risk of death in HCC patients was elevated with the increase of RiskScore (Figures 1(e) and 1(f)).

### 3.2. Validation of the Prognostic Model in the ICGC Cohort

**Figure 2:**
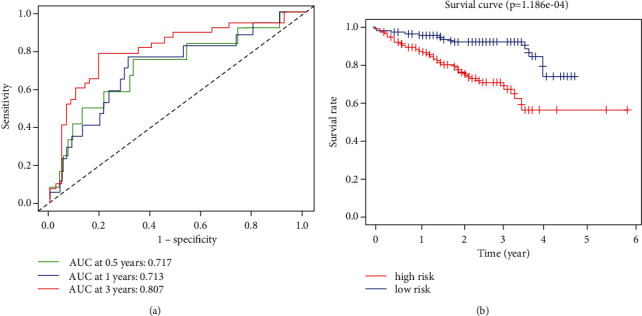
Validation of the prognostic model in ICGC cohort. (a) The ROC curves in ICGC cohort. (b) Comparison of survival data between the two groups.

The accuracy of the prognostic model was validated in 231 HCC samples from the ICGC cohort. The values of AUC at 0.5-, 1-, and 3-year survival were 0.717, 0.713, and 0.807 in the ICGC cohort, respectively (Figure 2(a)), confirming the reliability of the proposed prognostic model. In addition, we also found that the prognosis in the high-risk group was significantly worse than that in the low-risk group in the ICGC cohort (Figure 2(b)). These results are consistent with those achieved in TCGA cohort.

### 3.3. Identification of the Gene Sets in the Low-Risk Group

**Figure 3:**
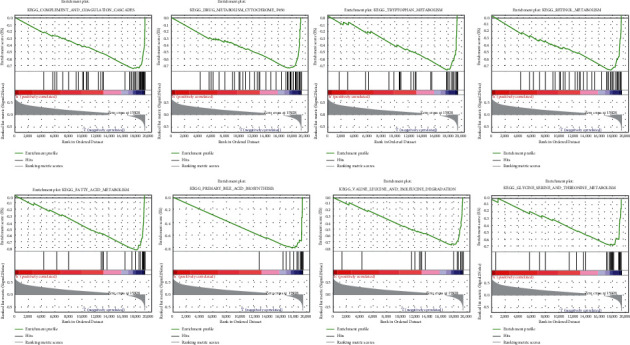
The gene sets in the low-risk group could be enriched in metabolic pathways.

According to the risk score in different groups, we detected the significant gene sets by GSEA. In the low-risk group, 8 gene sets were found (FDR (*q* value)<0.001), including COMPLEMENT_AND_COAGULATION_CASCADES, DRUG_METABOLISM_CYTOCHROME_P450, TRYPTOPHAN_METABOLISM, RETINOL_METABOLISM, FATTY_ACID_METABOLISM, PRIMARY_BILE_ACID_BIOSYNTHESIS, VALINE_LEUCINE_AND_ISOLEUCINE_DEGRADATION, and GLYCINE_SERINE_AND_THREONINE_METABOLISM (Figure 3). In the high-risk group, no gene set was identified (FDR (*q* value) <0.05)

### 3.4. The RiskScore Could be an Independent Prognostic Indicator

**Figure 4:**
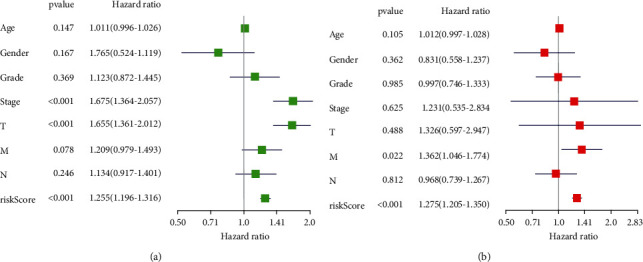
The relationship between RiskScore and patients' clinicopathological characteristics. Univariate cox regression analysis (a) and multivariate cox regression analysis (b) of patients' clinicopathological features.

We analyzed the relationship between the RiskScore and patients' clinicopathological characteristics (age, gender, histological grade, clinical stage, and TNM). Univariate Cox regression analysis of clinicopathological features showed that *P* values of stage, T stage, and RiskScore were <0.001 and HR was >1 (Figure 4(a)). Multivariate Cox regression analysis of clinicopathological features revealed that *P* values of RiskScore and M stage were <0.05 and HR was >1 (Figure 4(b)).

### 3.5. The Correlation of RiskScore with Patients' Clinicopathological Characteristics

**Figure 5:**
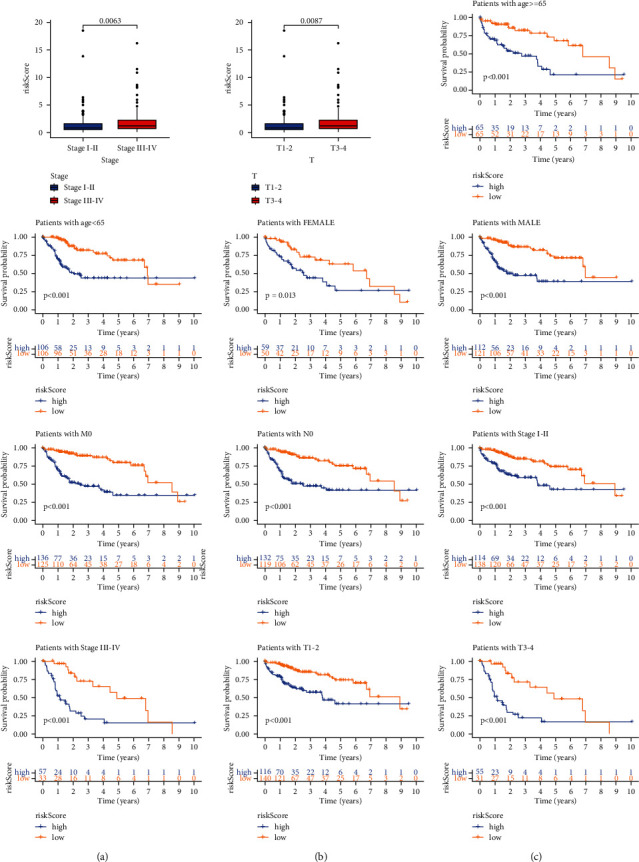
(a)The correlation of RiskScore with patients' clinicopathological characteristics. (b) The distribution of RiskScore in the two groups. (c) The RiskScore could predict the survival of patients with different ages, genders, and TNM stages.

We found that there were significant differences in T stage between the high-risk and low-risk groups (Figures 5(a) and 5(b)). The survival rate was significantly different in different ages, genders, and TNM stages (Figure 5(c)).

### 3.6. Relationship between RiskScore and ImmuneScore

**Figure 6:**
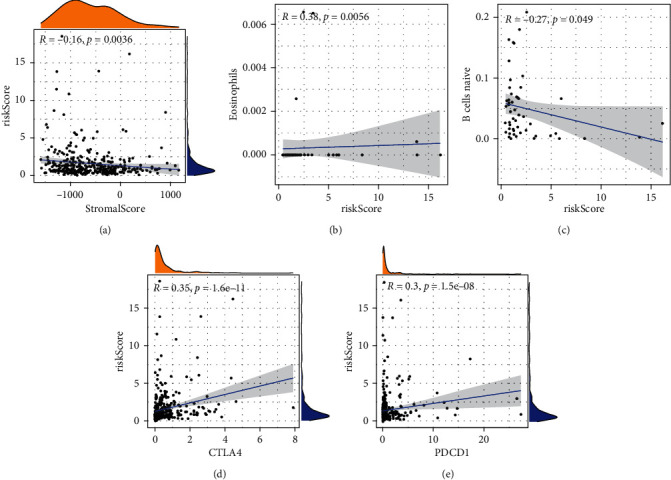
The relationship between RiskScore and ImmuneScore. (a) The RiskScore could be significantly negatively correlated with StromalScore. (b, c) The RiskScore could be positively correlated with the number of eosinophils and that could be significantly negatively correlated with the number of naïve B cells. (d, e) The RiskScore could be significantly positively correlated with the expression levels of CTLA4 and PDCD1.

We estimated the ImmuneScore of each patient by “estimate” package. Besides, we estimated the infiltration of immune cells in each patient and selected 52 samples with significant differences. We found that there was a significant negative correlation between RiskScore and StromalScore (Figure 6(a)). We also noted that there was a positive correlation between RiskScore and the number of eosinophils, as well as was a negative correlation between RiskScore and the number of naive B cells (Figures 6(b) and 6(c)). Finally, we found a positive correlation between RiskScore and the expression levels of immune checkpoint inhibitors (CTLA4 and PDCD1) (Figures 6(d) and 6(e)).

## 4. Discussion

At present, HCC is recognized as a type of cancer with a poor prognosis worldwide, and its effective treatment has markedly attracted scholars' attention. To date, several clinicians have attempted to develop the prognostic models for HCC. To our knowledge, the histological grade influences the treatment and prognosis of HCC patients. In the present study, we differentiated histological grades of HCC, identified DEGs in the two groups, constructed a prognostic model using DEGs, and verified it in the external database.

We downloaded HCC data from the TCGA database. We employed the univariate and multivariate Cox regression analyses, as well as the LASSO regression to establish the prognostic model. After verification of the proposed model using data downloaded from ICGC cohort, we found that the model was highly reliable, and it was revealed that the prognosis in the high-risk group was significantly worse than that in the low-risk group. Next, we explored the correlation of RiskScore with patients' clinicopathological characteristics, and we found that the RiskScore could be used as an independent prognostic factor, which further confirmed the reliability of our model. The microenvironment is a complex and dynamic system involving extracellular matrix (ECM) components, soluble factors, and stromal cells, whose distribution and composition vary in space and time [12]. The present study revealed that there was a significant negative correlation between StromalScore and RiskScore. Therefore, regulating the expressions of some genes in our model to cause epigenetic changes in stromal cells may be a new idea for the treatment of HCC. Eosinophils can secrete a variety of substances to affect tumor cells [13]. Carretero et al. pointed out that eosinophils orchestrate cancer rejection by normalizing tumor vessels and enhancing infiltration of CD8+ T cells [14]. Productive humoral responses require that naive B cells and their differentiated progeny move among distinct microenvironments [15]. We, in the current research, found the significant correlation between RiskScore and the number of eosinophils and naive B cells. The role of expression levels of PDCD1 and CTLA4 in immunotherapy has been widely studied. Our study found that there was a significant positive correlation between RiskScore and the expression levels of PDCD1 and CTLA4.

Gao et al. demonstrated that enhanced expression of TXNRD1 is associated with advanced tumor progression and metastasis of HCC [16]. Kudin et al. pointed out that the increased expression of TXNRD1 is associated with generalized epilepsy in human [17]. Fu et al. found that TXNRD1 is an unfavorable prognostic factor for patients with HCC [18]. Sun et al. reported that ANXA10 promotes the progression of perihilar cholangiocarcinoma and facilitated metastasis by promoting the epithelial-mesenchymal transition (EMT) process via the PLA2G4A/PGE2/STAT3 pathway [19]. Hung et al. demonstrated that Cul4A could regulate the degradation of ANXA10 through interaction with ANXA10 and ubiquitination in lung cancer cells [20]. Neoplasms mainly arise from a single cell of origin, and tumor progression results from acquired genetic variability within the original clone, allowing sequential selection of more aggressive sublines [21]. Huffmann et al. confirmed that a transmembrane protein, TMEM41B, is required for infection by members of the Flaviviridae [22]. Moretti et al. found that TMEM41B is a novel regulator of autophagy and lipid mobilization [23]. Zhao et al. demonstrated that ALAS1 has antitumor effects on colorectal cancer cells [24]. Peyer et al. described ALAS1 as a new direct target of the bile acid-activated nuclear receptor farnesoid X receptor [25].

Our model was established based on the results of statistical analysis [26]. Although the model was validated in the external database and its reliability was confirmed, utilization of further advanced statistical analyses can improve its reliability. We still need to verify the relationships between the 7 genes in the model by biological experiments, so as to find the potential relationship between the expression levels of these genes and the histological grade of HCC. Due to the limitations of data collection from public databases, it is highly essential to perform a prospective research to further confirm the proposed prognostic model. The proposed model could accurately predict the prognosis of HCC patients, assisting clinicians to study different histological grades of HCC.

## Data Availability

The datasets used and/or analyzed during the current study are available from the corresponding author upon request.

## Abbreviations:

HCC: Hepatocellular carcinoma
FPKM: Fragments per kilo base million
TCGA: The Cancer Genome Atlas
ICGC: International Cancer Genome Consortium
GTF: Gene transfer format
DEGs: Differentially expressed genes
GSEA: Gene set enrichment analysis.
